# Telemedicine Prescribing by US Mental Health Care Providers: National Cross-Sectional Survey

**DOI:** 10.2196/63251

**Published:** 2025-03-11

**Authors:** Mollie R Cummins, Julia Ivanova, Hiral Soni, Zoe Robbins, Brian E Bunnell, Esteban López, Brandon M Welch

**Affiliations:** 1 College of Nursing, University of Utah, 10 S 2000 East, Salt Lake City, UT, 84112-5880, United States, 1 8015859740; 2 Doxy.me, Inc, Charleston, SC, United States; 3 Department of Psychiatry and Behavioral Neurosciences, Morsani College of Medicine, University of South Florida, Tampa, FL, United States; 4 Biomedical Informatics Center, Public Health and Sciences, Medical University of South Carolina, Charleston, SC, United States

**Keywords:** telemedicine, telehealth, telemental, provider, professional, experience, attitude, opinion, perception, perspective, prescribing, prescription, drug, pharmacology, pharmacotherapy, pharmaceutic, pharmaceutical, medication, mental health, digital health, informatics, buprenorphine, ketamine, cross sectional, survey, questionnaire

## Abstract

**Background:**

In the postpandemic era, telemedicine continues to enable mental health care access for many people, especially persons living in areas with mental health care provider shortages. However, as lawmakers consider long-term telemedicine policy decisions, some question the safety and appropriateness of prescribing via telemedicine, and whether there should be requirements for in-person evaluation, especially for controlled substances.

**Objective:**

Our objective was to assess US telemental health care provider perceptions of comfort and perceived safety in prescribing medications, including controlled substances, via telemedicine.

**Methods:**

We conducted a web-based, cross-sectional survey of US telemental health care providers who prescribe via telemedicine, using nonprobability, availability sampling of a national telehealth research panel from February 13 to April 28, 2024. We used descriptive statistics, visualization, and thematic analysis to analyze results. We assessed differences in response distribution by health care provider licensure type (physician vs nonphysician) and specialty (psychiatry vs nonpsychiatry) using the Mann-Whitney *U* test.

**Results:**

A total of 115 screened and eligible panelists completed the survey. Overall, participants indicated high levels of comfort with prescribing via telemedicine, with 84% (102/115) of health care providers indicating they strongly agree with the statement indicating comfort in prescribing medications via telemedicine. However, participants indicated less comfort in prescribing if they have never seen a patient in person, or if the patient is located out-of-state. Most participants indicated they can safely prescribe controlled substances via telemedicine, without having previously provided care to a patient in person. However, 14.8% (17/115) to 19.1% (30/115) of health care providers (by schedule) felt that they could rarely or never safely prescribe controlled substances. There were some differences in perception of comfort and safety by licensure and specialty. Among controlled substance schedules, participants indicated the least perceived safety with schedule IV medications, and the most safety with schedule II and III medications.

**Conclusions:**

These health care providers were highly comfortable prescribing both scheduled and unscheduled medications via telemedicine. Comfort and perceived safety with telemedicine prescribing varied somewhat by licensure type (physician vs nonphysician) and specialty (psychiatry vs nonpsychiatry). Perceived safety varied moderately for scheduled medications (controlled substances), especially for schedule IV and V medications. Participants indicated use of adaptive strategies to prescribe safely depending upon the clinical context. In ongoing efforts, we are analyzing additional survey results and conducting qualitative research related to telemedicine prescribing. A strong understanding of prescriber perspectives and experience with telemedicine prescribing is needed to support excellent clinical practice and effective policy making in the United States.

## Introduction

During the COVID-19 public health emergency, the need for mental health services increased and telemedicine enabled critical access to care [1,2]. In the postpandemic era, telemedicine continues to enable mental health care access for many people, especially persons living in areas with mental health care provider shortages [3]. However, as lawmakers consider long-term policy decisions related to telemedicine, some have raised questions about the safety and appropriateness of prescribing via telemedicine, and whether there should be requirements for in-person evaluation, especially for controlled substances.

Before the US COVID-19 public health emergency declaration, most health care providers lacked telemedicine prescribing experience due to limited telemedicine adoption. Additionally, telemedicine-based prescribing of controlled substances was restricted during the prepandemic era, in compliance with the 2008 Ryan Haight Online Pharmacy Consumer Protection Act (Ryan Haight Act). Pursuant to the Ryan Haight Act, the US Drug Enforcement Agency (DEA) required that health care providers conduct an in-person evaluation of patients, before prescribing controlled substances via telemedicine. This measure was intended to prevent health care providers from prescribing potentially harmful medications with only minimal and inadequate online interaction with a patient. During the US public health emergency declaration, temporary policy flexibilities enabled controlled substance prescribing without the requirement of an in-person evaluation. In part, this enabled crucial access to buprenorphine for opioid use disorder treatment [4,5]. Now, consumers and stakeholders are calling for new policy that enables continued access to mental health care and substance use treatment, including medication-based treatment, via telemedicine [6]. The challenge is to develop evidence-based policy that supports safety while enabling critical health care access.

Current evidence is inconclusive but suggests that prescribing patterns can differ when care is provided via telemedicine versus in-person care. A 2023 study at a single institution showed that orthopedic patients are prescribed higher doses (milligram morphine equivalent) of opioids via video telemedicine than during in-person visits. McCabe et al [7] found that telemedicine is used less frequently than in-person visits for prescribing antibiotics. However, other studies have shown little or no difference in the prescribing patterns associated with telemedicine use [8,9]. These varied findings likely relate to differences in the clinical context for prescribing, including patient and health care provider characteristics, system-level factors, or the specific medications and their unique requirements for appropriate initiation and monitoring (and whether those requirements can be met via telemedicine). Evidence supporting safety and quality of telemedicine prescribing in mental health care is scarce, and also likely to be context dependent. However, a recent, large cohort study of health care claims data found no difference in safety or quality for telemedicine-based treatment versus in-person treatment of opioid use disorder, including medication-based opioid use disorder treatment [10].

Current health care provider perspectives and practices related to prescribing via telemedicine for mental or behavioral health care are not well-characterized. However, a better understanding of telemental health care provider’s perspectives and experiences related to prescribing is needed to inform appropriate telemedicine program design, identify key research questions, pursue clinical practice guidelines, and develop curricula for professional educational programs. The perspectives and experience of practicing telemental health care providers should also inform policy decisions, such as the currently pending US DEA rules governing telemedicine prescribing of buprenorphine for opioid use disorder. The purpose of this study was to assess the perspectives of US telemental health care providers related to their comfort and perceived safety in prescribing medications, including controlled substances, via telemedicine.

## Methods

### Study Design

We conducted a web-based, cross-sectional survey of telemental health providers who prescribe via telemedicine, using nonprobability, availability sampling of a telehealth research panel from February 13 to April 28, 2024.

### Survey

The web-based survey, administered using Qualtrics, was created by the research team and designed to elicit perspectives and practices of US mental or behavioral health care providers related to prescribing via telemedicine. The survey consisted of 8 sections: informed consent (1 question), verification of eligibility (5 questions), demographic and practice information (13 questions), comfort and safety (6 questions), health history (7 questions), physical assessment and diagnostic testing (7 questions), issuing a prescription (8 questions), and legal and regulatory environment (8 questions). A copy of the survey is provided in Multimedia Appendix 1.

This study is focused on a subset of the survey items, including Likert-scale items that measured agreement with statements indicating comfort with prescribing in varied scenarios, including in person (no telemedicine), via telemedicine, and via telemedicine with and without previous in-person care. We also analyzed Likert-scale items that measured agreement with statements of ability to safely prescribe medications, by US DEA scheduling. Recognizing that safe prescribing can depend upon individual patient characteristics and contextual factors aside from telemedicine use or pharmacotherapy, we designed the Likert scale to measure how often medications of a given schedule can be prescribed safely via telemedicine. Optional open-ended items prompted participants to elaborate upon their responses.

### Survey Development

The interdisciplinary research team, which includes expertise in telemedicine, clinical pharmacology, and advanced practice nursing in primary care, developed the initial survey based on a literature review. A small group of telemedicine practitioners reviewed an early draft of the survey and provided input on its content, format, and relevance for clinical practice. We then refined the survey according to their input. Before initiating data collection, we informally pretested and modified the survey within the research team, then formally pretested the survey with the target audience of 5 prescribing telemental health providers located in Utah and North Carolina who met the inclusion criteria. All 5 individuals who pretested the survey indicated that they strongly agree with the following statements: (1) the content and wording of the survey is appropriate; (2) the survey is easy to understand; (3) the survey is free of errors; (4) the time required to complete the survey is reasonable; and (5) the time required to complete the survey would be reasonable if the participants are compensated. Pretesters completed the survey in mean 11.9 (SD 4.24) minutes and recommended US $25‐$50 as compensation for completing the survey. The survey was configured to assign completion codes, so that the process of participant compensation, which requires collecting additional personal information, could be managed separately from data collection.

### Sampling and Recruitment

We implemented nonprobability, availability sampling of a telehealth research panel. We calculated a target sample size of 382 for 95% CI. We currently lack precise data describing the number of US health care providers who (1) provide mental health care and (2) prescribe via telemedicine. Therefore, we based the target sample size Health Resources and Services Administration estimates of the total number of US mental health care providers in prescribing roles [11].

We recruited survey participants from the TelehealthEngage Research Panel, a panel of 7134 telemedicine users who have consented to be contacted about opportunities to participate in research. TelehealthEngage includes individuals from 49 states who are active users of Doxy.me, a commercial telemedicine platform. We invited all TelehealthEngage panelists who identified as a health professional in mental health, psychiatry, family practice, internal medicine, general practice, neurology, integrative medicine, or unknown fields or who identified as a physician, hospitalist, physician assistant (PA), nurse practitioner, or nurse. Recruitment was initiated on February 13, 2024, and closed on April 28, 2024. We divided our 2 recruitment waves over the course of 3 days each: the first invite was sent to 2343 unique panelists and the second to 3490. We sent a reminder email to any panelist who had not opened the email invitation (1491 in the first wave and 3200 in the second wave) after 3 weeks. The survey was closed out 2 weeks after the last invitation reminder was sent out. We required responses to all categorical or numeric survey items to complete the survey and be compensated for participation with a US $50 gift card.

### Ethical Considerations

This study was reviewed and approved by the BRANY (Biomedical Research Alliance of New York) Institutional Review Board (IRB00010793). To protect the anonymity of participants, we configured Qualtrics to assign a random completion code to each completed survey. Upon survey completion, a code was displayed to participants along with instructions to visit a separate Qualtrics survey to submit the code and contact information for compensation. In this way, we separated participants’ responses from their contact information. We stored the data in a secure environment with access limited to essential study personnel. Participants were compensated for participation with a US $50 gift card.

### Analysis

We analyzed all completed surveys. We calculated descriptive statistics and frequencies, visualized response distributions, and aggregated free-text responses for qualitative analysis. Further, we assessed group differences in response distributions by licensure type (physician vs nonphysician) and specialty type (psychiatric vs nonpsychiatric) for items using Mann-Whitney *U* tests. We performed all statistical analysis using SPSS (version 29; IBM Corp) and performed a qualitative, thematic analysis of open-ended items using MAXQDA (MAXQDA - Distribution by VERBI GmbH). The results of the quantitative analyses informed the way we organized codes from the thematic analysis [12,13]. Further, 1 author coded the qualitative responses from surveys using the entire participant response as the unit of analysis. After 3 iterations of coding, a codebook was developed and honed with the help of all authors. We assigned each response one or more codes, and so the total number of codes exceeds the number of responses.

## Results

### Participation and Response

In total, 350 TelehealthEngage panelists accessed the survey. Of those, 336 (96%) completed all eligibility screening questions, and 121 (36%) of those screened were eligible. Further, 115/121 (95%) of screened and eligible panelists completed the survey. There was no missing data in completed surveys given the requirement that all items be completed, except optional free text fields. Only 4 surveys were initiated but not completed, so we did not analyze the data or individual items for nonresponse bias. See Table 1 illustrating study participation results.

**Table 1.** Participant count by stage.

Stage	Value, n
Accessed survey	350
Completed consent and screening items	336
Eligible	121
Initiated survey	119
Completed survey	115

### Participant Characteristics

**Figure 1.**
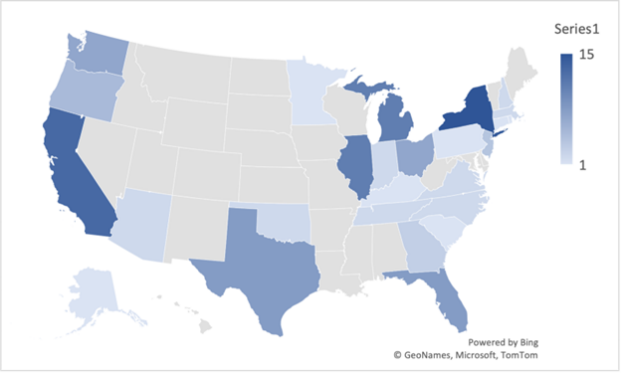
Geographic distribution of participants (N=115). Created using Bing Maps [14] and published under the platform's limited license [15].

Demographic characteristics of the participants are summarized in Table 2 and Figure 1. The participants were largely White, non-Hispanic, and female, with a mean age of 51.2 (SD 12.4) years and primarily physicians and advanced practice nurses in smaller-sized practices. The most common specialties were psychiatry or psychiatry specialties. However, 28 (24.3%) indicated nonpsychiatry specialties. Geographically, the participants were distributed across 26 American states (Figure 1).

**Table 2.** Demographic and practice characteristics of participants (N=115).

Characteristic	Values, n (%)
Race	
American Indian or Alaska Native	1 (0.9)
Asian	9 (7.8)
Black or African American	8 (7)
Native Hawaiian or Pacific Islander	0 (0)
White	83 (72.2)
More than 1 race	5 (4.3)
Other race	2 (1.7)
Prefer not to answer	7 (6.1)
Unknown	0 (0)
Hispanic or Latino	
Hispanic or Latino	8 (7)
Not Hispanic or Latino	97 (84.3)
Prefer not to answer	6 (5.2)
Unknown	4 (3.5)
Gender	
Male	46 (40)
Female	66 (57.4)
Nonbinary or third gender	0 (0)
Prefer not to say	3 (2.6)
Licensure	
Doctor of medicine or doctor of osteopathic medicine	69 (60)
Advanced practice registered nurse or physician assistant	40 (34.8)
Doctor of philosophy clinical psychologist	4 (3.)
Pharmacist	0 (0)
Other	2 (1.74)
Percent of clients seen via telehealth	
None (0%)	0 (0)
Few (1%‐24%)	10 (8.7)
Some (25%‐49%)	23 (20)
Most (50%‐74%)	33 (28.7)
Almost all (75%‐99%)	29 (25.2)
All (100%)	20 (17.4)
Practice size	
Independent	53 (46.1)
Small group (2‐5 health care providers)	29 (25.2)
Mid-size (6‐15 health care providers)	20 (17.4)
Large group practice (16+ health care providers)	13 (11.3)
Setting^a^	
Academic	6 (5.2)
Community	47 (40.9)
Hospital	4 (3.5)
Clinic	58 (50.4)
School	0 (0)
Corrections	0 (0)
Federally qualified health center	4 (3.5)
Digital health care	17 (4.8)
Other	6 (5.2)
Specialty	
Psychiatry	63 (54.8)
Addiction medicine or psychiatry	6 (5.2)
Child and adolescent psychiatry	14 (12.2)
Geriatric psychiatry	4 (3.5)
Forensic psychiatry	0 (0)
Consultation liaison psychiatry	0 (0)
Family practice	11 (9.6)
Internal medicine	6 (5.2)
Pediatrics	3 (2.6)
Other	8 (7)
Experience in specialty (years)	
0‐5	24 (20.9)
6‐10	20 (17.4)
11‐15	23 (20)
16+	48 (41.7)
Experience in telehealth (years)	
0‐3.7 (since onset of COVID-19 pandemic)	70 (60.9)
3.7‐10	39 (33.9)
11‐15	3 (2.6)
16+	3 (2.6)

a Participants could select more than 1 descriptor.

### Main Findings

**Figure 2.**
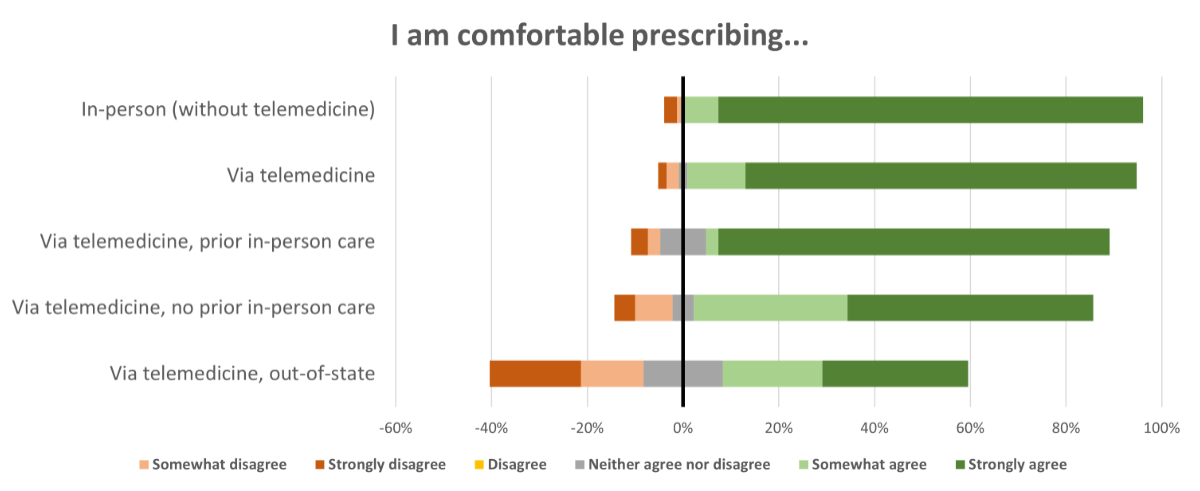
Participant agreement with statements of comfort in prescribing via telemedicine (N=115).

Overall, participant health care providers indicated high levels of comfort with prescribing via telemedicine, with 84% (102/115) of participants indicating they strongly agree with the statement indicating comfort in prescribing medications via telemedicine. Only 5 (4.3%) participants somewhat or strongly disagreed. However, participants indicated less comfort in prescribing if they have never seen a patient in person, or if the patient is located out-of-state. Figure 2 shows a visualization of the response distribution; detailed descriptive statistics are provided in Multimedia Appendix 2.

Further, 50% (58/115) of participants responded to the optional question “Please tell us more about the situations in which you feel comfortable or not comfortable prescribing.” We found 3 general categories of comments related to comfort: comments related to feeling uncomfortable (1 instance), comfortable (17 instances), or more or less comfortable depending on the specific situation (conditionally comfortable, 36 instances).

**Figure 3.**
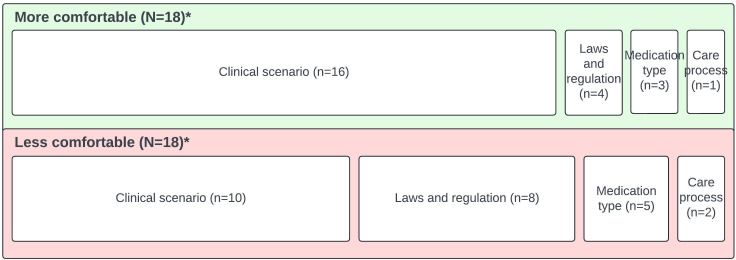
Condition types where health care providers feel conditionally more or less comfortable prescribing via telemedicine. *N refers to codes of more or less comfortable, depending upon conditions. n refers to code counts for the condition type. The total number of codes for condition type exceeds that of codes for comfort.

The singular response that a participant health care provider unequivocally did not feel comfortable prescribing over telemedicine was in regard to controlled substances: “Will not prescribe classified drugs or anything for ADHD online.” Health care providers who noted they unequivocally felt comfortable prescribing over telemedicine (17 instances) also included some explanation of prescribing situations regarding laboratories and assessments, laws and regulations, and types of medication: “All but one of my current patients I’ve met in person, but my practice is now solely telemedicine. I do my best to assess the patient’s condition, personality, and response. I think I am as comfortable prescribing online as in person.” The majority (36 instances) of health care providers reported their comfort levels depended upon certain conditions. The approximate distribution of condition types is depicted in Figure 3. Health care providers were more (18 codes) or less comfortable (18 codes) due to reasons relating to the clinical scenario (including patient characteristics or behavior); laws and regulations; types of medications; and the care process (characteristics of the patient’s care plan or care delivery including specific types of visits, assessments, and laboratories). Numerous health care providers noted how an initial in-person intake (9 codes) or working with established patients (9 codes) affected their experiences and comfort: “I felt comfortable after having an in person visits [sic] with the patient before telemedicine. I do a physical exam on all patients during the first visit snd sm [sic] not sure I feel comfortable prescribing without that initial physical encounter.” Complete results of thematic analysis for this item are provided in Multimedia Appendix 3.

**Figure 4.**
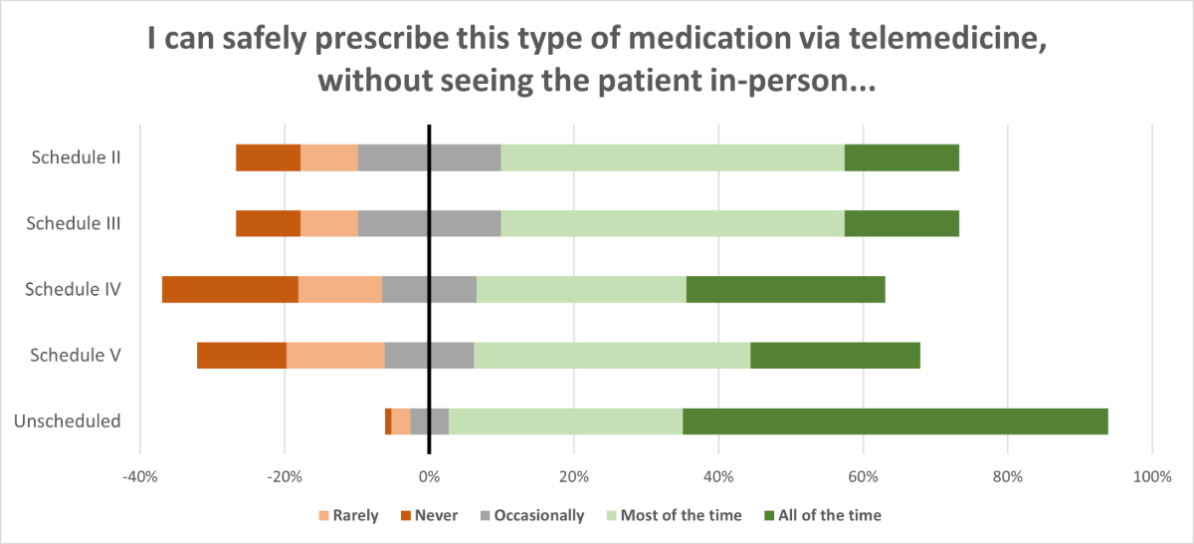
Perceived safety of telemedicine-based prescribing for medication types, by US Drug Enforcement Agency drug scheduling (N=115).

For telemedicine-based prescribing of controlled substances, the results are visualized in Figure 4 (detailed descriptive statistics in Multimedia Appendix 2). Most participants indicated that they can safely prescribe controlled substances via telemedicine, without having previously provided care to a patient in person. However, 14.8%‐19.1% of health care providers (by schedule) felt that they could rarely or never safely prescribe controlled substances. Among controlled substance schedules, participants indicated the least perceived safety with schedule IV medications, and the most safety with schedule II and III medications.

Further, 44% (51/115) of participants responded to the optional question “Tell us more about the types of medications you feel comfortable prescribing and/or monitoring via telemedicine, and the circumstances in which you feel an in-person assessment is appropriate…” We found 2 general categories of comments related to comfort: comfortable (33 instances) and not comfortable (33 instances).

**Figure 5.**
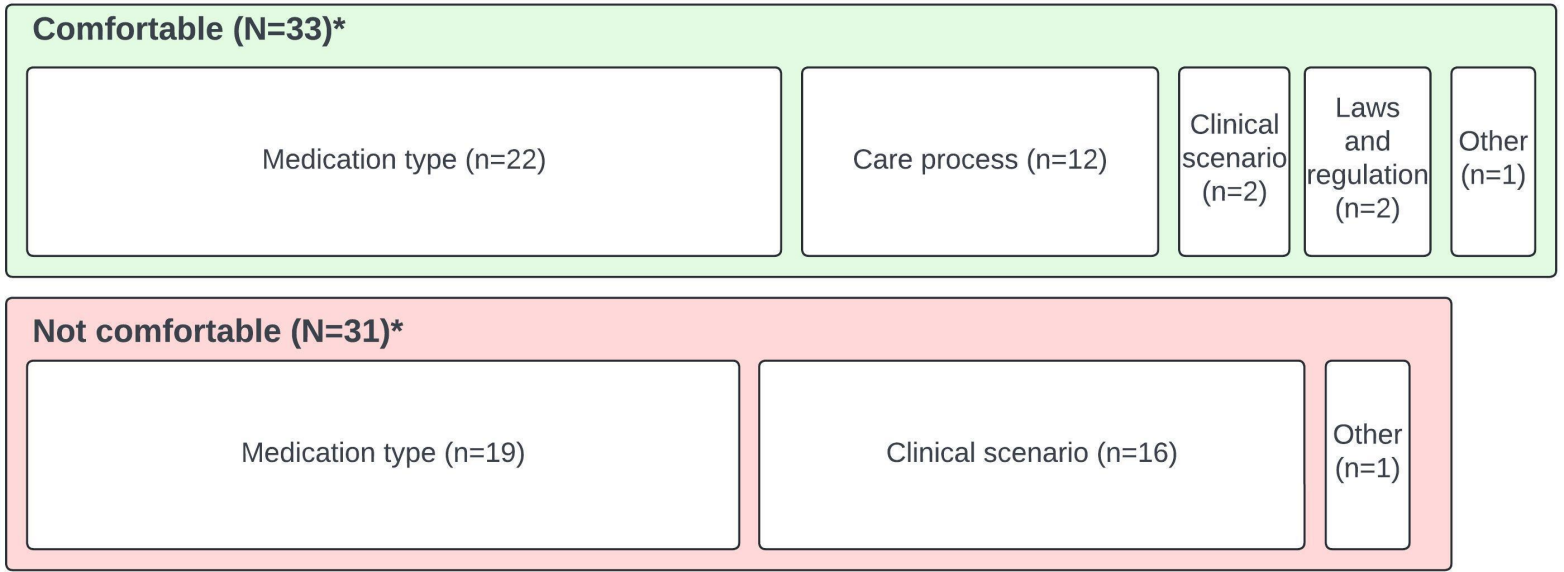
Comments on comfort with specific types of medications and need for in-person appointments. *N refers to incidents and n refers to code counts. The total number of codes exceeds that of incidents.

As depicted in (Figure 5), health care providers reported they felt comfortable (33 instances) prescribing certain types of medications (22 codes), prescribing with given certain care activities or processes (12 codes), in relation to laws and regulations (2 codes), in certain clinical scenarios (2 codes), and other (1 code). Further, 1 health care provider explained their requirements regarding medication types for prescribing over telemedicine: “No analgesics prescribed (no opiates, etc.) but I do prescribe stimulants for psychiatric reasons (ADHD, post-concussive pathology, as an adjunct for depression). My use of benzos is very conservative. Otherwise, I rarely prescribe anything but ‘standard’ psych meds–anti-depressants, mood stabilizers, non-benzo anxiolytics, at times anti-psychotics.” Health care providers reported not feeling comfortable (31 instances) due to prescribing certain types of medications (19 codes), certain clinical scenarios (16 codes), and preferring in-person prescribing (1 code). Further, 1 health care provider noted how certain clinical scenarios and medication types affect their comfort: “I would only feel uncomfortable prescribing controlled substances virtually if I was [sic] concerned about other illicit substance use or elevated blood pressure/heart rate. I would want to see [the] patient in person for vital signs and UDS [urine drug screen]. The [state controlled substance database] allows us to monitor controlled prescriptions our patients have obtained.” Complete results of thematic analyis for this item are provided in Multimedia Appendix 3.

### Findings by Licensure and Specialty

Table 3 summarizes the results of the Mann-Whitney *U* tests to determine whether response differed according to licensure type (physician vs nonphysician) or specialty (psychiatry vs nonpsychiatry). We found that comfort in prescribing differed by licensure type for in-person prescribing (no use of telemedicine, *U*=1857.5; *P*=.005; common language effect size [CL]=0.59), with physicians indicating stronger baseline agreement that they are comfortable with in-person prescribing (mean rank of 61.92 vs a mean rank of 52.12 for nonphysicians). We also found that physicians differed from nonphysicians in perception of their ability to safely prescribe schedule V medications (*U*=2018.5; *P*=.01; CL=0.64), with visualization showing that physicians indicated greater perceived safety (mean rank 64.25 vs 48.62). There were no other statistically significant differences in the distribution of responses to statements of comfort in prescribing by licensure type.

**Table 3.** Difference in distribution of responses by licensure type and specialty (N=115).

Item	Licensure (physician or nonphysician)			Specialty (psychiatric or nonpsychiatric)		
	*U(SD)*	*P* value	CL^a^	*U*	*P* value	CL^a^
I am comfortable prescribing medications…						
In person (no telemedicine)	1857.5	.005^a^	0.59	1216	.98	0.50
Telemedicine	1796.5	.08	0.57	1292.5	.47	0.53
Telemedicine, prior in person	1666	.50	0.52	1215.5	.98	0.50
Telemedicine, no prior in person	1435	.34	0.45	1571	.01	0.64
Telemedicine, out-of-state	1651.5	.71	0.52	1039	.23	0.43
I can safely prescribe this type of medication via telemedicine, without seeing the patient in person:						
Schedule II	1701.5	.50	0.54	1409.5	.19	0.58
Schedule III	1562	.88	0.49	880	.02	0.36
Schedule IV	1749	.34	0.55	1487.5	.07	0.61
Schedule V	2018.5	.01[Table-fn T3_FN1]	0.64	1063	.30	0.44
Unscheduled medications	1774.5	.22	0.56	1419.5	.13	0.58

a CL: common language effect size.

By specialty, we found that the distribution of responses for prescribers from psychiatric specialties differed from that of nonpsychiatric specialty prescribers for comfort in prescribing via telemedicine when there has been no previous in-person care (*U*=1571; *P*=.01; CL=0.64), with prescribers from psychiatric specialties indicating more comfort (mean rank 62.06 vs 45.39). Prescribers from psychiatric specialties also differed for the perceived safety of prescribing schedule III medications (*U*=880; *P*=.02; CL=0.36), with visualization showing that prescribers from psychiatric specialties indicated lower perceived safety for this type of medication (mean rank 54.11 vs 70.07). Every participant with a specialty of addiction medicine or psychiatry (n=6) indicated that they can safely prescribe schedule III medications either “always” or “almost always.” However, the number of addiction medicine or psychiatry specialists was not sufficient to support testing of group differences.

## Discussion

### Principal Findings

In this survey of US telemental health care providers, we examined prescribing telemental health care provider perceptions of comfort and safety in prescribing medications via telemedicine. Largely White, non-Hispanic, female, and middle-aged, the participants’ demographic characteristics reflect the known demographics of the US mental health care workforce [16]. Licensure type and medical specialty varied. Participant practice settings were primarily individual or small group, and nonacademic. Most participants were physicians, advanced practice registered nurses, or PAs, and psychiatry was the most common specialty. Geographically, participants were fairly distributed within the United States. However, there was no representation of the mountain west.

Overall, we found that telemental health care providers were very comfortable prescribing via telemedicine. However, they were slightly less comfortable when prescribing to a patient not previously seen in person, and even less comfortable prescribing to an out-of-state patient. Psychiatry specialists expressed more comfort than nonpsychiatry specialists with providing care to patients not previously seen in person. The effect size was moderate for these differences. Qualitative analysis showed that individual prescriber comfort varied depending on the context, including the clinical scenario, types of medications being prescribed, laws and regulations, and care process. This finding is consistent with prior research indicating that a health care provider’s discomfort in prescribing over telemedicine appears to be tempered by access to additional information regarding the patient and situation such as access to laboratories, in-person follow-up, and coordination of care [17,18]

We further examined the participants’ perspectives on their ability to safely prescribe unscheduled medications as well as scheduled medications, which are controlled substances. Participants indicated that they are highly comfortable prescribing unscheduled medications. However, they varied in their comfort prescribing controlled substances. While participants in this study indicated they can usually prescribe schedule II-V medications safely, a minority indicated that they can rarely or never prescribe these medications safely. Variation in comfort by DEA scheduling may relate more to prescriber familiarity and experience with those medications, than with safety issues. For example, we found that physicians felt more comfortable than nonphysicians in prescribing schedule V medications, with a moderate effect size. Examples of schedule V medications are pregabalin (Lyrica) and diphenoxylate or atropine (Lomotil). Given most psychiatric medications fall under schedules II-IV, this could reflect a broader medical practice of physicians in comparison to other licensure types. Psychiatry specialists were less comfortable than nonpsychiatry specialists in prescribing schedule III medications with no prior in-person care. Examples of schedule III medications include buprenorphine (Suboxone), testosterone, and ketamine. It is plausible that nonpsychiatry specialists, unlikely to prescribe buprenorphine or ketamine in their practices, may have based their response on other medications within schedule III, medications with which they are more familiar.

All addiction psychiatry or medicine specialists indicated a high level of comfort in prescribing schedule III medications. It is plausible that addiction specialists have cultivated practices that enhance safety and reduce harm, increasing their sense of safety with schedule III medications. A typical addiction specialist may enhance safety through a policy of checking controlled substance databases, requiring random drug toxicology screens and securing permission to speak with patients’ families. Prescribers who routinely treat addiction also often have the advantage of intersecting with integrative partners such as substance use disorder treatment centers, court appointed case managers, etc, which may enhance their sense of comfort and safety in prescribing. Addiction specialists may be better prepared to learn that a patient has diverted medications, sought additional prescribers, or knowingly taken drugs or prescriptions outside of their prescribed regimen, and could be less likely to fear these circumstances in the context of treatment.

In open-ended responses, participants described substantial tailoring of their individual prescribing practices and decision-making according to the clinical context and their comfort level in prescribing. This finding is indicative of individual adaptation of practice to perceived risks and uncertainties. Currently, best practices for prescribing via telemedicine are general in nature, and health care providers rely on local practice settings’ rules and processes for guidance [19]. Individual prescribers may differ in how they approach virtual and hybrid (virtual and in-person) care, but likely adapt their practice within their comfort level through measures that include coordination of care, in-person follow-up appointments, requirements for assessments and laboratories, and compliance with laws and regulations [20]. Ultimately, our findings show that telemental health care providers prescribe over telemedicine at their discretion, in the interest of their patients’ health, and according to their individual sense of comfort. Our findings also initiate that prescribers implement care activities and processes that enhance safety.

### Limitations

This study used a nonprobability sampling approach; we recruited a convenience sample from a research panel of telemental health care providers. The sample size and study design do not support the generalizability of findings to all US mental health care providers. However, these findings reflect the perspectives of a large, national sample of these health care providers. TelehealthEngage panelists are users of the Doxy.me telemedicine platform which is heavily used by solo and small clinic practices, and this is reflected in the participant demographics. Perspectives may differ for health care providers who are part of large enterprise settings or academic settings. Additionally, we may not have captured nuanced considerations of prescribing safety or the conditions under which prescribers feel comfortable prescribing via telemedicine. Medications within a single controlled substance schedule are used for different purposes and have different safety considerations. For example, the use of telemedicine to prescribe ketamine is currently highly controversial, as the mental health community continues to debate the appropriateness of using a medication that induces a dissociative state without the support of an in-person therapist or guide.

We also recognize that telemedicine implementation models vary and likely influence perspectives on prescribing comfort and safety. For example, a prescriber who provides telemental health care in the context of a hub-and-spoke model, where a registered nurse or medical assistant augments virtual care with an in-person assessment, may have perceptions of greater comfort and safety in prescribing than a prescriber practicing without such a model. In ongoing work, we are conducting qualitative research that entails interviewing prescribers to more fully elucidate this information. However, this survey study allowed us to first characterize the overarching perspectives of a larger number of prescribers.

### Conclusion

We conducted a national, cross-sectional survey of US telemental health providers to assess their comfort and perceived safety in prescribing medications, including controlled substances, via telemedicine. The participants included physicians, advanced practice nurses, PAs, and clinical psychologists. These health care providers were highly comfortable prescribing both scheduled and unscheduled medications via telemedicine. Comfort and perceived safety with telemedicine prescribing varied somewhat by licensure type (physician vs nonphysician) and specialty (psychiatry vs nonpsychiatry). Perceived safety varied moderately for scheduled medications (controlled substances), especially for schedule IV and V medications. Participants indicated use of adaptive strategies to prescribe safely depending upon the clinical context. In ongoing efforts, we are analyzing additional survey results and conducting qualitative research related to telemedicine prescribing. A strong understanding of prescriber perspectives and experience with telemedicine prescribing is needed to support excellent clinical practice and effective policy making in the United States.

## Supplementary material

10.2196/63251 Multimedia Appendix 1 Survey of telemental health providers and informed consent form.

10.2196/63251 Multimedia Appendix 2 Descriptive statistics.

10.2196/63251 Multimedia Appendix 3 Results of thematic analysis of open-ended questions.

## ABBREVIATIONS

BRANY: Biomedical Research Alliance of New York
CL: common language effect size
DEA: Drug Enforcement Agency
PA: physician assistant
