# Supplementary material for: Telemedicine Prescribing by US Mental Health Care Providers: National Cross-Sectional Survey
Source: JMIR Form Res. 2025 Mar 11;9:e63251. doi: 10.2196/63251 (PMC11939023; doi:10.2196/63251)
Supplement: Multimedia Appendix 1 [file formative-v9-e63251-s001.pdf]

Informed Consent

Information to Consider Before Taking Part in this Research

Study Title: Provider perceptions and experiences of telemedicine

Study #: IRB00010793

Overview: You are being asked to take part in a research study. The information in this document provides basic information about the study and should help you to decide if you would like to participate.

Study Staff: This study is being led by Julia Ivanova, PhD who is a research associate at Doxy.me Inc. This person is called the Principal Investigator. Other approved research staff may act on behalf of the Principal Investigator.

Study Details: This study is being conducted by Doxy.me Inc. The purpose of this study is to understand providers' perspectives on providing healthcare and prescribing medication via telemedicine. The survey will be administered via Qualtrics and should take about 10-20 minutes to complete. Data collected will be de-identified, removing respondents' IP addresses and location data, prior to any analysis. At the end of the survey, respondents will be asked whether they are interested in participating in a 30-60 minute-long, semi-structured, paid virtual interview: if willing, respondents will provide contact information.

Respondents will be asked for verbal consent to record the interview for the purposes of transcription and analysis. Audio and video recordings will be deleted after transcription and all interview data will be de-identified prior to analysis.

Participants: You are being asked to take part because you are a telemedicine healthcare professional, over the age of 18 years, and an English speaker.

Voluntary Participation: Your participation is voluntary. You do not have to participate and may stop your participation at any time. There will be no penalties or loss of benefits or opportunities if you do not participate or decide to stop once you start. Deciding to participate or not to participate will not affect your job status or your relationship with doxy.me. There is no cost to participate in this study.

Benefits, Compensation, and Risk: We do not know if you will receive any benefit from your participation. There is no cost to participate. Compensation will be provided in the form of a $50 egift card upon completion of the survey. For those respondents who complete the hour-long semi-structured interview, a $75 egift card will be sent out. This research is considered minimal risk. Minimal risk means that study risks are the same as the risks you face in daily life. There is a risk of loss of confidentiality, though, researchers will take all feasible measures to protect against such a risk. As there is no feasible risk for injury during the survey or interview, Doxy.me, Inc. does not cover compensation for injury.

Confidentiality: All survey data is de-identified for research purposes. Anyone with the authority to look at your records must keep them confidential. If you take part in this study, you will be assigned a unique subject code to help protect your privacy. Your study records and study samples will be labeled with this code that does not directly identify you. The study site staff securely stores the linking code between your name and study information. It is possible, although unlikely, that unauthorized individuals could gain access to your responses because you are responding online. Confidentiality will be maintained to the degree permitted by the technology used. No guarantees can be made regarding the interception of data sent via the Internet. However, your participation in this online survey and potential virtual interview involves risks similar to a person's everyday use of the Internet. If you complete and submit an anonymous survey and later request your data be withdrawn, this may or may not be possible as the researcher may be unable to extract anonymous data from the database.

Contact Information

If you have any questions, concerns or complaints about this study, contact Julia Ivanova, PhD at julia.ivanova@doxy.me or 602-561-8861.

If you have any questions about your rights as a research subject or complaints regarding this research study, or you are unable to reach the research staff, you may contact a person independent of the research team at the Biomedical Research Alliance of New York Institutional Review Board at 516-318-6877. Questions, concerns or complaints about research can also be registered with the Biomedical Research Alliance of New York Institutional Review Board at www.branyirb.com/concerns-about-research. The IRB is a

committee that reviews research studies to help protect the rights and welfare of study subjects.

Dr. Julia Ivanova is a paid employee of Doxy.me Inc., a commercial telemedicine platform company.

You can print a copy of this consent form for your records.

I freely consent to take part in this study. I understand that by proceeding with this survey, I am agreeing to take part in research, and I am 18 years of age or older.

- I consent.
- I do not consent.

Did not consent

Thank you for your interest. Please direct any questions to Dr. Julia Ivanova, julia.ivanova@doxy.me or 602-561-8861

Verify Eligibility

Part A. Verifying your eligibility to participate

First, we'll ask you some questions to verify if you are eligible to participate in the study. If you are **not eligible** to participate, you'll see a message indicating the survey has ended. If you are **eligible**, you will be able to proceed with the survey. Upon completing the survey, we'll ask for your e-mail address so that we can send you an e-gift card to compensate you for your participation. We'll also ask you if you'd like to participate in an interview for additional compensation.

Let's get started...

Are you currently licensed to prescribe medications in the U.S.?

- ◯ No
- ◯ Yes

Do you provide mental health care services?

- No
- Yes

How frequently do you prescribe medications during telehealth/ virtual visits?

- Never
- Sometimes
- About half the time
- Most of the time
- Always

What is your age, in years?

You are ineligible to participate

Thank you for your interest, but you are not eligible to participate in this study. Please direct any questions to Dr.

Julia Ivanova, julia.ivanova@doxy.me or 602-561-8861

Demographic and Practice Information

Thank you for verifying your eligibility to participate! Let's proceed with the rest of the survey...

Part B. Demographic and Practice Information

What is your race?

- ○ American Indian or Alaska Native
- ○ Asian
- ○ Black or African American
- ○ Native Hawaiian or Other Pacific Islander
- ○ White
- ○ More than one race
- ○ Other Race
- ○ Prefer not to Answer
- ○ Unknown

Do you have an Hispanic or Latino ethnic background?

- Hispanic or Latino
- Not Hispanic or Latino
- Prefer not to answer
- Unknown

What is your gender?

- Male
- Female
- Non-binary / third gender
- Prefer not to say

Which best describes your licensure?

- MD or DO
- Advanced practice nurse/ Nurse practitioner/ APRNPhysician’s Assistant
- PhD clinical psychologist
- Pharmacist
- [ ] Other

What percent of your clients do you see via telehealth/ virtual visits?

- None (0%)
- Few (1-24%)
- Some (25-49%)
- Most (50-74%)
- Almost all (75-99%)
- All (100%)

Which best describes the size of your telemedicine practice setting?

- Independent practice
- Small group practice (2-5 providers)
- Mid-size practice (6-15 providers)
- Large group practice (16+)

Which best describes the nature of your telemedicine practice setting? (Please check all that apply.)

- Academic
- Community
- Hospital
- Clinic

- ☐ School
- ☐ Corrections
- ☐ Federally Qualified Health Center
- ☐ Digital health care (services delivered via an application or web-based system independent of a traditional health care setting)
- ☐ Other

What is your specialty?

- ○ Psychiatry
- ○ Addiction Medicine or Addiction Psychiatry
- ○ Child & Adolescent Psychiatry
- ○ Geriatric Psychiatry
- ○ Forensic Psychiatry
- ○ Consultation Liaison Psychiatry
- ○ Family Practice
- ○ Internal Medicine
- ○ Pediatrics
- ○ [ ] Other

Years of experience in specialty:

- ○ 0-5 years
- ○ 6-10 years

- 11-15 years
- 16 or more years

How long have you been practicing with telemedicine?

- 0-3.7 years (since the onset of the COVID-19 pandemic)
- 3.7-10 years
- 11-15 years
- 16 or more years

What is the most advanced degree you've obtained?

- Master's degree
- Doctoral degree
- Other

In which state do you primarily practice?

Comfort and Safety

Part C. Comfort and Safety

Please indicate your agreement with the following statements about comfort with prescribing:

	Strongly disagree	Somewhat disagree	Neither agree nor disagree	Somewhat agree	Strongly agree
I am comfortable prescribing medications **in-person.**	○	○	○	○	○
I am comfortable prescribing medications **via telemedicine.**	○	○	○	○	○
I am comfortable prescribing medications **via telemedicine if I have previously met with the patient in-person.**	○	○	○	○	○
I am comfortable prescribing medications **via telemedicine, even if I have never previously met with the patient in-person.**	○	○	○	○	○

	Strongly disagree	Somewhat disagree	Neither agree nor disagree	Somewhat agree	Strongly agree
I feel comfortable prescribing medication **via telemedicine, to a patient located in another state.**	○	○	○	○	○

Please tell us more about the situations in which you feel comfortable or not comfortable prescribing... (optional)

I can safely prescribe this type of medication **via telemedicine, without seeing the patient in-person**:

	N/A; I don't prescribe this type of medication	Never	Rarely	Occasionally	Most of the time	All of the time
**Schedule II** medications (i.e. methadone (Methadose, Dolophine), dextroamphetamine-amphetamine (Adderall), hydromorphone (Dilaudid), methylphenidate (Ritalin))	○	○	○	○	○	○
**Schedule III** medications (i.e. buprenorphine (Suboxone), testosterone, ketamine)	○	○	○	○	○	○
**Schedule IV** medications (i.e. alprazolam (Xanax), zolpidem (Ambien), tramadol(Ultram))	○	○	○	○	○	○
**Schedule V** medications (i.e. pregabalin (Lyrica), diphenoxylate/atropine (Lomotil)	○	○	○	○	○	○
Medications that are **not scheduled** (i.e. duloxetine (Cymbalta), paroxetine (Paxil), certraline (Zoloft))	○	○	○	○	○	○

I can safely **monitor** this type of medication use via telemedicine:

	N/A; I don’t prescribe this type of medication	Never	Rarely	Occasionally	Most of the time	All of the time
**Schedule II** medications (i.e. methadone (Methadose, Dolophine), dextroamphetamine-amphetamine (Adderall), hydromorphone (Dilaudid), methylphenidate (Ritalin))	○	○	○	○	○	○
**Schedule III** medications (i.e. buprenorphine (Suboxone), testosterone, ketamine)	○	○	○	○	○	○
**Schedule IV** medications (i.e. alprazolam (Xanax), zolpidem (Ambien), tramadol(Ultram))	○	○	○	○	○	○
**Schedule V** medications (i.e. pregabalin (Lyrica), diphenoxylate/atropine (Lomotil)	○	○	○	○	○	○

	N/A; I don't prescribe this type of medication	Never	Rarely	Occasionally	Most of the time	All of the time
Medications that are **not scheduled** (i.e. duloxetine (Cymbalta), paroxetine (Paxil), certraline (Zoloft))	○	○	○	○	○	○

Tell us more about the types of medications you feel comfortable prescribing and/ or monitoring via telemedicine, and the circumstances in which you feel an in-person assessment is appropriate... (optional)

Health History

Part D. Health History

To assess the patient's **drug allergies** when prescribing via telemedicine, I...

	Never	Rarely	Occasionally	Most of the time	All of the time
Ask the patient.	○	○	○	○	○
Review a record of the patient's allergies.	○	○	○	○	○

What type(s) of records do you consult to determine the patient's **drug allergies**? (Check all that apply).

- ☐ Patient-completed form
- ☐ Electronic health record (e.g., Epic, Cerner)
- ☐ Personal health record (e.g., patient portal, google, myhealth)
- ☐ Paper health record or medical record
- ☐ Other

To assess the patient's **current medications** when prescribing via telemedicine, I...

	Never	Rarely	Occasionally	Most of the time	All of the time
Ask the patient.	○	○	○	○	○

	Never	Rarely	Occasionally	Most of the time	All of the time
Review a record of the patient's medications.	○	○	○	○	○

What type(s) of records do you consult to determine the patient's **current medications**? (Check all that apply).

- ☐ Patient-completed form
- ☐ Electronic health record (i.e. Epic, Cerner)
- ☐ Personal health record (i.e. patient portal, google, myhealth)
- ☐ [ ] Other

To assess the patient's **medical history** when prescribing via telemedicine, I...

	Never	Rarely	Occasionally	Most of the time	All of the time
Ask the patient.	○	○	○	○	○
Review a record of the patient's medical history.	○	○	○	○	○

What type(s) of records do you consult to determine the patient's **medical history**? (Check all that apply).

- ☐ Patient-completed form
- ☐ Electronic health record (i.e. Epic, Cerner)
- ☐ Personal health record (i.e. patient portal, google, myhealth)
- ☐ Other

Physical Assessment and Diagnostic Testing

Part E. Physical Assessment and Diagnostic Testing

How often are you unable to prescribe via telemedicine because a physical examination is necessary?

- ◯ Never
- ◯ Sometimes
- ◯ About half the time
- ◯ Most of the time
- ◯ Always

If physical examination is necessary, how often do you use each of the following to meet the need for physical examination?

	Never	Rarely	Occasionally	Most of the time	All of the time
Conduct the physical examination during a separate, in-person visit.	◯	◯	◯	◯	◯
Refer to another health care provider.	◯	◯	◯	◯	◯
Refer to another health care provider within my organization.	◯	◯	◯	◯	◯
Refer to another health care provider outside my organization.	◯	◯	◯	◯	◯
Transfer care of the patient to another health care provider.	◯	◯	◯	◯	◯
Maintain responsibility for care of the patient, but consult or coordinate with another health care provider for physical examination.	◯	◯	◯	◯	◯

	Never	Rarely	Occasionally	Most of the time	All of the time
Use my clinical judgement in safely prescribing without a physical examination.	○	○	○	○	○

Feel free to provide clarification to your answers regarding physical assessments in the context of prescribing over telemedicine. (optional)

If laboratory testing is necessary in order to safely and appropriately prescribe a medication, are you able to arrange for appropriate testing?

- ○ Never
- ○ Sometimes
- ○ About half the time
- ○ Most of the time

○ Always

If laboratory testing (or other diagnostic testing) is necessary, how often do you use each of the following to meet the need?

	Never	Rarely	Occasionally	Most of the time	All of the time
Refer to another health care provider for care.	○	○	○	○	○
Refer to another health care provider **within my organization**.	○	○	○	○	○
Refer to another health care provider **outside my organization**.	○	○	○	○	○
Transfer care of the patient to another health care provider.	○	○	○	○	○
Maintain responsibility for care of the patient, but consult or coordinate with another health care provider for laboratory testing.	○	○	○	○	○

	Never	Rarely	Occasionally	Most of the time	All of the time
Use my clinical judgement in safely prescribing without laboratory testing.	○	○	○	○	○

Feel free to provide clarification to your answers regarding laboratory testing in the context of prescribing over telemedicine. (optional)

Issuing a prescription

Part F. The process of issuing a prescription

In order to issue a prescription to a patient via telemedicine, I do the following. (Please check all that apply)

- ☐ Call a pharmacy
- ☐ Email a pharmacy
- ☐ Fax a pharmacy
- ☐ Send an e-script to a pharmacy using a computer-based system or platform
- ☐ Mail a paper prescription
- ☐ [ ] Other

Please indicate your level of agreement with the following statements about the process of issuing a prescription to a patient via telemedicine:

	Strongly disagree	Somewhat disagree	Neither agree nor disagree	Somewhat agree	Strongly agree
The process of issuing a prescription is cumbersome.	○	○	○	○	○
The process of issuing a prescription is easy and straightforward.	○	○	○	○	○
I **have access** to computer-based systems or tools that help me issue prescriptions.	○	○	○	○	○

	Strongly disagree	Somewhat disagree	Neither agree nor disagree	Somewhat agree	Strongly agree
I **would like** access to computer-based systems or tools that help me issue prescriptions.	○	○	○	○	○

Have you ever had a patient who experienced an adverse event **from a prescription provided over telemedicine**? (An adverse event is any undesirable experience associated with the use of a medical product in a patient).

- ○ No
- ○ Yes
- ○ I don't know
- ○ Prefer not to answer

In your opinion, could that adverse event(s) have been avoided if the prescription had been provided during an in-person visit?

- ○ Definitely not
- ○ Probably not
- ○ Might or might not
- ○ Probably yes

- Definitely yes

When prescribing via telemedicine… I can provide care that is **consistent with widely accepted clinical practice guidelines**.

- Strongly disagree
- Somewhat disagree
- Neither agree nor disagree
- Somewhat agree
- Strongly agree

When prescribing via telemedicine… **I follow an established safety or quality procedure**.

- Never
- Sometimes
- About half the time
- Most of the time
- Always

Please feel free to clarify your answers regarding guidelines and safety/ quality procedures in the context of prescribing

over telemedicine. (optional)

Legal & Regulatory Environment

Part G. Legal & Regulatory Environment

Please indicate your agreement with the following statements about prescribing via telemedicine:

	Strongly disagree	Somewhat disagree	Neither agree nor disagree	Somewhat agree	Strongly agree
I feel **confident** that I understand how to comply with current law and regulation related to prescribing via telemedicine.	○	○	○	○	○

	Strongly disagree	Somewhat disagree	Neither agree nor disagree	Somewhat agree	Strongly agree
I feel **confident** that I can stay up-to-date with changes in law and regulation related to prescribing via telemedicine.	○	○	○	○	○
I feel **uncertain** about compliance with current law and regulation when prescribing via telemedicine.	○	○	○	○	○
I have **difficulty understanding** whether I can prescribe for patients who are located out-of-state.	○	○	○	○	○

In general, do you feel your state's current prescribing laws **as they relate to telemedicine** are:

- ○ Overly restrictive
- ○ Appropriate
- ○ Not sufficiently restrictive
- ○ I don't know them well enough to express an opinion.

Feel free to provide clarification to your answers regarding the legal and regulatory environment in the context of prescribing over telemedicine. (optional)

To ensure that I'm up-to-date on compliance, I depend upon guidance from: (Check all that apply)

- ☐ My health care organization
- ☐ Online telehealth or telemedicine resource center
- ☐ Specific web site(s)
- ☐ Search on the internet
- ☐ Newsletter
- ☐ Word of mouth

How important is it to **your practice** that **prescribing via telemedicine** continue?

- ○ Not at all important
- ○ Slightly important

- Moderately important
- Very important
- Extremely important

How important to **your patients** that **prescribing via telemedicine** continue?

- Not at all important
- Slightly important
- Moderately important
- Very important
- Extremely important

Are there specific sources of guidance related to compliance that you depend upon? (optional)

Thank you for Participating

Thank you for participating in the survey. To receive your e-gift card, please **copy or make note of the completion code** below, and proceed here: https://utahnursing.co1.qualtrics.com/jfe/form/SV_1HyrHdd9

Completion Code

${e://Field/RandomID}

Powered by Qualtrics
