# Supplementary material for: Telemedicine Prescribing by US Mental Health Care Providers: National Cross-Sectional Survey
Source: JMIR Form Res. 2025 Mar 11;9:e63251. doi: 10.2196/63251 (PMC11939023; doi:10.2196/63251)
Supplement: Multimedia Appendix 2 [file formative-v9-e63251-s002.docx]

**Table S1.** Participant agreement with statements of comfort in prescribing via telemedicine, absolute and percentage frequencies (N=115).

| I am comfortable prescribing medications… | | Strongly disagree | | Somewhat disagree | | Neither agree nor disagree | | Somewhat agree | | Strongly agree | |
| --- | --- | --- | --- | --- | --- | --- | --- | --- | --- | --- | --- |
|  |  | % | n | % | n | % | n | % | n | % | n |
| 1 | in-person. | 2.61 | 3 | .87 | 1 | .87 | 1 | 6.96 | 8 | 88.70 | 102 |
| 2 | via telemedicine. | 1.74 | 2 | 2.61 | 3 | 1.74 | 2 | 12.17 | 14 | 81.74 | 94 |
| 3 | via telemedicine if I have previously met with the patient in-person. | 3.48 | 4 | 2.61 | 3 | 9.57 | 11 | 2.61 | 3 | 81.74 | 94 |
| 4 | via telemedicine, even if I have never previously met with the patient in-person. | 4.35 | 5 | 7.83 | 9 | 4.35 | 5 | 32.17 | 37 | 51.3 | 59 |
| 5 | via telemedicine, to a patient located in another state. | 19.13 | 22 | 13.04 | 15 | 16.52 | 19 | 20.87 | 24 | 30.43 | 35 |

**Table S2.** Perceived safety of telemedicine-based prescribing safety for medication types, by U.S. Drug Enforcement Agency (DEA) drug scheduling, absolute and percentage frequencies (N=115).

| I can safely prescribe this type of medication via telemedicine, without seeing the patient in-person: | | N/A; I don't prescribe this type of medication | | Never | | Rarely | | Occasionally | | Most of the time | | All of the time | |
| --- | --- | --- | --- | --- | --- | --- | --- | --- | --- | --- | --- | --- | --- |
|  |  | % | n | % | n | % | n | % | n | % | n | % | n |
| 1 | Schedule II medications (i.e. methadone (Methadose, Dolophine), dextroamphetamine-amphetamine (Adderall), hydromorphone (Dilaudid), methylphenidate (Ritalin)) | 12.17 | 14 | 7.83 | 9 | 6.96 | 8 | 17.39 | 20 | 41.74 | 48 | 13.91 | 16 |
| 2 | Schedule III medications (i.e. buprenorphine (Suboxone), testosterone, ketamine) | 47.83 | 55 | 11.30 | 13 | 6.96 | 8 | 7.83 | 9 | 17.39 | 20 | 8.70 | 10 |
| 3 | Schedule IV medications (i.e. alprazolam (Xanax), zolpidem (Ambien), tramadol(Ultram)) | 10.43 | 12 | 6.09 | 7 | 13.04 | 15 | 19.13 | 22 | 35.65 | 41 | 15.65 | 18 |
| 4 | Schedule V medications (i.e. pregabalin (Lyrica), diphenoxylate/atropine (Lomotil) | 29.57 | 34 | 8.70 | 10 | 9.57 | 11 | 8.70 | 10 | 26.96 | 31 | 16.52 | 19 |
| 5 | Medications that are not scheduled (i.e. duloxetine (Cymbalta), paroxetine (Paxil), certraline (Zoloft)) | 0.87 | 1 | 0.87 | 1 | 2.61 | 3 | 5.22 | 6 | 32.17 | 37 | 58.26 | 67 |
