# Supplementary material for: Telemedicine Prescribing by US Mental Health Care Providers: National Cross-Sectional Survey
Source: JMIR Form Res. 2025 Mar 11;9:e63251. doi: 10.2196/63251 (PMC11939023; doi:10.2196/63251)
Supplement: Multimedia Appendix 3 [file formative-v9-e63251-s003.docx]

The Codebook from the question “**Please tell us more about the situations in which you feel comfortable or not comfortable prescribing**” includes 58 (N=58) unique entries. Four entries did not answer the question or could not be coded. The three codes are broken down into their respective subcodes and sub-subcodes. Due to the nature of the coding, subcodes may not add up to the parent code.

| Subcode | Sub-subcode | Example Quote | n |
| --- | --- | --- | --- |
|  |  |  |  |
| **DOES NOT feel comfortable** | | | **1** |
| Will not prescribe any scheduled drugs via telemedicine |  | "Will not prescribe classified drugs or anything for ADHD online" | 1 |
|  |  |  |  |
| **DOES feel comfortable** | | | **17** |
| Generally feels comfortable prescribing over telemedicine |  | "All but one of my current patients I've met in person, but my practice is now solely telemedicine. I do my best to assess the patient's condition, personality, and response.  I think I am as comfortable prescribing online as in person." | 5 |
| In relation to specific visit types, assessments, labs |  | "If I need lab work or vital signs, my patients and I are comfortable in obtaining those and making them part of documentation." | 4 |
| In relation to state borders, laws, and regulations |  | "I feel comfortable prescribing medication in person or via telehealth. Seeing a psychiatric patient in person is not necessary for me unless they are experiencing suicidal intentions/plans, homicidal ideations/intentions/plans or psychosis. I chose to not see patients via telehealth who are suicidal, homicidal or psychotic. I feel comfortable prescribing out of state for non controlled medications but currently I only have a license to prescribe medication in Florida." | 1 |
| In relation to types of medications |  | "I feel comfortable with most psychiatric medications, including antidepressants and mood stabilizers. Telemedicine makes it difficult to appreciate extrapyramidal symptoms with antipsychotics. As it pertains to stimulants and benzodiazepines, having the patient obtain vitals themselves can be a challenge." | 3 |
|  |  |  |  |
| **Comfort DEPENDS on the situation** | | | **36** |
| LESS Comfortable |  | | *18* |
|  | In relation to specific visit types, assessments, labs (Care process) | "I do not like prescribing meds when I do not have the proper labs." | 2 |
|  | In relation to state borders, laws, and regulations (Laws and regulation) | "Many of my patients I have never met in person only virtually and I do feel comfortable prescribing medicines to them. I do not feel comfortable prescribing medicines out of state as that is outside of the bounds of my IL medical license." | 8 |
|  | In relation to types of medications (Medication type) | "I feel very uncomfortable with prescribing Scheduled medications to a patient that I have not seen in person, or across state lines. I will not prescribe outside DEA regulations." | 5 |
|  | Patient-dependent reasons (situational reasons, symptoms, or diagnoses) (Clinical scenario) | "I felt comfortable after having an in person visits with the patient before telemedicine. I do a physical exam on all patients during the first visit snd sm not sure I feel comfortable prescribing without that initial physical encounter" | 10 |
| MORE Comfortable |  | | *18* |
|  | In relation to specific visit types, assessments, labs (Care process) | "All but one of my current patients I've met in person, but my practice is now solely telemedicine. I do my best to assess the patient's condition, personality, and response.  I think I am as comfortable prescribing online as in person." | 1 |
|  | In relation to state border, laws, and regulations (Laws and regulation) | "I only prescribe medication to patients in state that no am licensed.  I prefer an in person visit before prescribing if at all possible." | 4 |
|  | In relation to types of medications (Medication type) | "It really depends on type of medications that is being prescribed. I do not normally prescribed any controlled substances via telemedicine unless has been stable on those meds and historical urine drug screens were appropriate.  Also with regards of antibiotics; I have prescribed based on symptoms and history of present illness.  Many pt with c/o dysuria I do not readily prescribe antibiotics and will have them get urine to lab asap.  Also so much more comfortable with prescribing those known to our practice as oppose to those we have never seen" | 3 |
|  | Patient-dependent reasons (situational reasons, health history) (Clinical Scenario) | "Comfortable if I know the person and have been treating, and then they move out of state for college or work to continue treating them" | 16 |

We found 51 (N=51) unique entries to the question “**Tell us more about the types of medications you feel comfortable prescribing and/or monitoring via telemedicine, and the circumstances in which you feel an in-person assessment is appropriate…**”. The two codes are broken down here by their subcodes. Due to the nature of the coding, subcodes may not add up to the parent code.

| Subcode | Example Quote | n |
| --- | --- | --- |
|  |  |  |
| **DOES NOT feel comfortable** | | **31** |
| In certain patient scenarios | "If I feel they are not consistent or forthcoming, or not willing to do blood pressure in front of me on screen" | 16 |
| Prescribing certain types of medications | "for controlled substances, req in person care first to stabilize and for genuine diagnosis then only recommend virtual follow up. Noncontrolled sub can be prescribed with virtual monitoring." | 19 |
| Prescribing in person preferred | "the question above is vague as to whether there has ever been face to face visits, so it makes it harder to answer. especially for initial prescribing, in person is far better" | 1 |
|  |  |  |
| **DOES feel comfortable** | | **33** |
| In certain patient scenarios (Clinical Scenario) | "for controlled substances, req in person care first to stabilize and for genuine diagnosis then only recommend virtual follow up. Noncontrolled sub can be prescribed with virtual monitoring." | 2 |
| Prescribing certain types of medications (Medication type) | "I do monitor their refills by the gov database to track appropriate refills and potential medications that can interact (ie I am prescribing a benzodiazepine and they didn‚Äôt tell me about the opioid prescription but I can see it). For benzos, I require a video visit and complete adherence with appointments. For suboxone, I require utox so they have to come into clinic to see the medical assistant. For stimulants, I am more limited as there is a high rate of substance dependence in the pts I treat, but the pts on them are people who are primarily sober. I ensure I have vitals for any pts on the above meds and that usually requires medical records that I review or for them to come into clinic to get vitals." | 22 |
| Prescribing with certain requirements/needs met (Care process) | "for controlled substances, req in person care first to stabilize and for genuine diagnosis then only recommend virtual follow up. Noncontrolled sub can be prescribed with virtual monitoring." | 12 |
| In relation to laws and regulations (Laws and regulation) | "Any breakdown in communication or compliance by the patient will require an in person visit. Any prescribing of scheduled medications will require at least one visit in person, per DEA regulations, outside of a national emergency (such as COVID pandemic)." | 2 |
| Other | "I prescribe Schedule 2 meds but only under my collaborative doctor's name" | 1 |
